# Biobrane™ for burns of the pubic region: minimizing dressing changes

**DOI:** 10.1186/s40779-018-0177-2

**Published:** 2018-08-26

**Authors:** Jia-Jun Feng, Jia Le See, Abby Choke, Adrian Ooi, Si Jack Chong

**Affiliations:** 1 Department of Plastic, Reconstructive and Aesthetic Surgery, Singapore General Hospital, Singapore, Singapore; 2 University of Auckland, Faculty of Medical and Health Sciences, Auckland, New Zealand

**Keywords:** Pubic, Thermal burns, Perineum, Dressing, Biobrane™

## Abstract

**Background:**

The pubic region is often involved in accidental hot water or soup-spill burns. Most of these wounds are superficial partial thickness burns. Due to their proximity to the urinary system, as well as vaginal and anal openings, these burns are easily contaminated. Daily dressings are routinely prescribed as the sole treatment. The cumbersome dressing process is uncomfortable and embarrassing for patients. Biobrane™ is a bilayered biosynthetic dressing. Its coverage of superficial partial thickness burns promotes wound healing and allows one-time application.

**Case presentations:**

We report two patients who suffered superficial dermal burns over their pubic region. One patient had 23% total body surface area (TBSA) burns over her lower abdomen, both thighs and pubic region. The second patient had 10% TBSA burns that involved her perineum and the medial sides of both thighs and buttocks. Both were managed with the standard resuscitation protocol in the initial phase. Their burn injuries were managed by shaving, Foley catheterization and Biobrane™ coverage. Their wounds healed uneventfully without complications. Full epithelization was achieved by post-operative day seven. Both patients consented to medical photography and academic publication.

**Conclusion:**

Shaving and catheterization improved the hygiene of the burns of the pubic area. The Biobrane™ method circumvents the need of regular dressing changes, eliminating the pain due to dressing changes and preserving patient dignity.

## Background

Pubic burns are commonly associated with upper thigh and lower abdominal wall burns. These burns are usually caused by accidental spills when patients are carrying a container of hot fluid. Due to the mechanism of burns and the coverage of clothing, pubic burns are almost always superficial partial thickness burns [1].

The management of these burns is routinely focused on the thighs and abdomen. For the pubic region, depending on the wound bed preparation and the available dressings, several dressing changes are required for burn wounds to heal in two to three weeks’ time. The dressing changes are often complicated by the presence of pubic hair and convoluted surface contours of this area. Difficult and frequent dressing changes lead to significant pain and discomfort for the patients. Furthermore, due to the sensitive nature of the area, it is inconvenient and embarrassing for patients to go through dressing changes, especially in an Asian context.

Biobrane™ is a biosynthetic dressing indicated for superficial partial thickness burns [2]. It is made of porcine dermal collagen-bonded nylon membrane on a silicon scaffolding. The collagen component initially adheres to fibrin on a clean wound surface and this adherence contributes to pain reduction. The silicone outer layer allows some water loss but prevents excessive water loss, thereby promoting desirable moist wound healing. Its transparency allows wound inspection [3]. Furthermore, it is highly pliable and stretchable, allowing for its successful application to various body parts with complex surface contours, including the ear [4] and buttock [5]. Additionally, this treatment avoids frequent dressing changes and allows wound epithelization within one to two weeks. These properties make it an ideal dressing for pubic burns, minimizing the pain and discomfort associated with daily dressing changes.

Our two cases support the claim of Smith and Nephews™ regarding healing within one to two weeks without donor site morbidity and without painful daily dressings using Biobrane™ for pubic burns. The study was approved by the Singheath Centralized Institutional Review Board (CIRB reference 2017/2444).

## Case presentations

### Case A

**Fig. 1:**
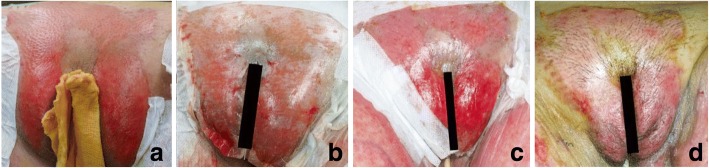
Pubic burns treated with Biobrane™. **a**. Preoperative photograph showing burns over pubic region; **b**. Intraoperative photograph after Biobrane™ application; **c**. Biobrane™ adhered well on POD 2. **d**. Full epithelization on POD 7

A 68-year-old woman with no prior medical problems sustained thermal burns when she spilled hot soup onto herself. She presented to the emergency department immediately. The initial assessment revealed 23% total body surface area (TBSA) superficial partial thickness burns involving the lower abdomen, bilateral thighs and pubic region including the mons pubis and labia majora (Fig. 1a).

She was started on fluid resuscitation, and a urinary bladder catheter was inserted for monitoring of fluid balance. She underwent burn scrub-down and Biobrane™ application 16 h after the burn.

Intraoperatively, the pubic hair was shaved, and the mons area was scrubbed down thoroughly. One piece of 10 cm × 10 cm Biobrane™ was applied and split in the middle of the lower half for a better fit for the labia majora and to keep the vestibule opening patent. It was secured by Hypafix™ superiorly to the lower abdominal wall and Vicryl Rapid™ 5–0 sutures inferiorly to the labia majora (Fig. 1b). Moist half-strength iodine gauze was then used to cover the Biobrane™.

On post-operative day two (POD 2), the Biobrane™ was well-adherent to the pubic region (Fig. 1c). The burns wounds were fully epithelized by POD 7 (Fig. 1c), allowing removal of the urinary bladder catheter, and the patient was subsequently discharged.

### Case B

**Fig. 2:**
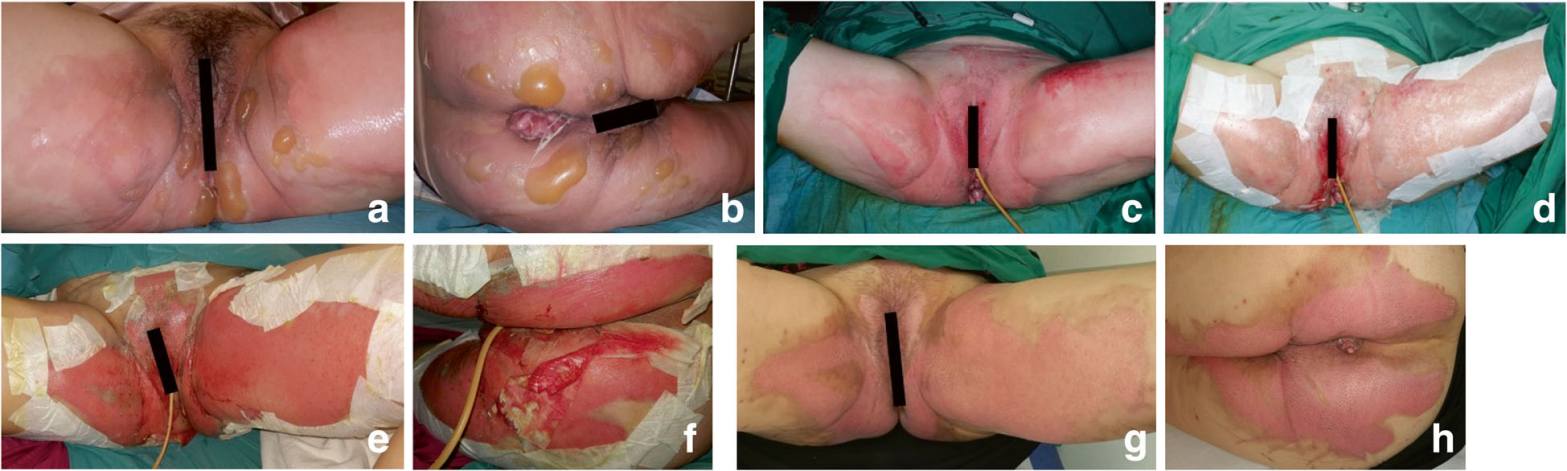
Patient with pubic region burns managed successfully with Biobrane™. **a**,**b**. Preoperative photograph showing burn wounds; **c**,**d**. Intraoperative photograph before and after Biobrane™ application; **e**,**f**. Biobrane™ adhered well on POD 2; **g**,**h**. Full epithelization achieved on POD 7

A 47-year-old woman with well-controlled hypertension and grade IV external hemorrhoids dropped a hot pot of soup on herself, scalding both her thighs, buttocks and perineum. She presented to the emergency department immediately. The clinical assessment showed a 10% TBSA partial thickness burn (mixture of superficial to mid dermal burns) involving the supra-pubic region, bilateral anterior thighs, perineum and bilateral buttocks and the labia majora and minora (Fig. 2a). She also suffered mucosal burns of her grade IV prolapsed hemorrhoids (Fig. 2b).

On admission, a Foley catheter was inserted to keep her affected areas clean. She underwent surgical scrub-down and Biobrane™ application 12 h after her burns.

She underwent burn scrub-down (Fig. 2c) and application of Biobrane™ similarly to the previous patient (Fig. 2d).

On POD 2, the Biobrane™ was noted to be well-adherent to the pubic wounds (Fig. 2e and f). On POD 7, the burn wounds had fully re-epithelized (Fig. 2g and h) and the urinary bladder catheter was removed. She was subsequently discharged on the same day.

## Discussion

We presented two cases of successful treatment of pubic burns with Biobrane™ coverage, made possible with Foley catheter placement and pubic hair removal to ensure good hygiene in a difficult-to-treat area.

The pliable and stretchable nature of Biobrane™ permits a seamless apposition of the dressing to the complex surface contour of the pubic region. Pubic hair shaving in both cases provided a smooth wound surface for optimal wound adherence of the Biobrane™. Urinary diversion by the catheter during initial complete bedrest aided Biobrane™ wound adherence and minimized urinary contamination.

There is evidence suggesting that the application of Biobrane™ may play a significant role in reducing rates of wound infection and wound desiccation [6–8]. The outcomes of our cases showed that the combination of Biobrane™ coverage after pubic hair removal and placement of a Foley catheter allowed complete Biobrane™ wound adherence in the pubic area with complex surface contours. The successful Biobrane™ application minimized wound infection and promoted wound healing, allowing prompt recovery in both patients.

Furthermore, the use of Hypafix™ and Vicryl Rapid™ suture to secure the Biobrane™ obviated the need for removal of stitches or staples after wound healing. For both patients, the Biobrane™ came off once the wounds healed in one week’s time without several dressing changes. The discomforts associated with dressing changes as well as suture and staple removal, were minimized. Furthermore, the patient’s dignity was probably more respected with this treatment regimen.

Admittedly, Biobrane™ application requires general anesthesia, and it comes with a greater cost than that of regular dressings. Additionally, most of these pubic burns heal in two to three weeks’ time despite the inconvenience of regular dressings. Therefore, the procedure should be considered only when patients require general anesthesia for Biobrane™ or skin graft coverage for extensive burns over other body parts.

## Conclusion

Biobrane™ dressing should be considered for superficial partial thickness burns of the pubic region, especially for patients who are undergoing general anesthesia for burn coverage of other body parts. With this approach, patients benefit from fast wound healing and their comfort and dignity are maximized.

## Abbreviations

POD: Post-operative day
TBSA: Total body surface area
